# Seroepidemiology of Toxoplasmosis among People Having Close Contact with Animals

**DOI:** 10.3389/fimmu.2015.00143

**Published:** 2015-04-28

**Authors:** Guo-Jie Brandon-Mong, Nurul Asma Anati Che Mat Seri, Reuben Sunil-Kumar Sharma, Hemah Andiappan, Tian-Chye Tan, Yvonne Ai-Lian Lim, Veeranoot Nissapatorn

**Affiliations:** ^1^Department of Parasitology, Faculty of Medicine, University of Malaya, Kuala Lumpur, Malaysia; ^2^Faculty of Veterinary Medicine, University Putra Malaysia, Selangor, Malaysia

**Keywords:** anti-*Toxoplasma* antibodies, IgG avidity, prevalence, risk factors, toxoplasmosis, people with animal-contact

## Abstract

A cross-sectional study was conducted to determine the seroepidemiology of *Toxoplasma* infection and its risk association among people having close contact with animals. A total of 312 blood samples were collected from veterinary personnel (veterinarian, technicians, and students) and pet owners from veterinary clinics and hospitals in the area of Klang Valley, Malaysia. About 4 cc of blood samples drawn from agreed participants were processed for measurement of anti-*Toxoplasma* IgG and IgM antibodies as well as avidity test of *Toxoplasma* IgG by ELISA I, II, and III kits. Meanwhile, the demographic profiles and possible risk factors of these participants were also recorded in the standardized data collection sheets. Overall seroprevalence of toxoplasmosis was observed in 62 (19.9%) participants being 7 (18.4%) in veterinarians, 15 (33.3%) in veterinary technicians, 29 (14.9%) in veterinary students, and 11 (31.4%) in pet owners. Of 19.9% *Toxoplasma* seropositive samples, 18.3% was positive for IgG antibody, 1.0% for IgM antibody, and 0.6% for both IgG and IgM antibodies. Of three different IgG avidity ELISA kits, ELISA III showed high avidity in all five seropositive samples (IgM and IgG/IgM antibodies) indicating chronic *Toxoplasma* infection which is consistent with no evidence of clinical toxoplasmosis diagnosed during the time of this study. Univariate analysis showed that age group, gender, study population, gardening, task performance, and working duration were significantly associated with *Toxoplasma* seropositivity. Further analysis by multivariate analysis using logistic regression showed that age group of ≥30 years old (OR = 0.34, 95% CI = 0.18–0.63, *p* = 0.001) and working or study duration of >10 years having close contact with animals (OR = 5.07, 95% CI = 1.80–14.24, *p* = 0.002) were identified as significant risks for *Toxoplasma* infection. Based on the results obtained, a comprehensive *Toxoplasma* screening and health surveillance program on toxoplasmosis should be implemented among people having close contact with animals in general and confirmed *Toxoplasma* seronegative individuals in particular to prevent seroconversion.

## Introduction

*Toxoplasma gondii* (*T. gondii*), an obligate intracellular protozoan parasite (a zoonotic pathogen) is capable of causing both the infection rate that affects approximately one-third of human populations worldwide and the disease burden of clinical toxoplasmosis in human. *Toxoplasma* infection can be transmitted via several routes in different host species (1). Many species of warm blooded animals can be infected including human and it was recognized by the National Institutes of Health, Bethesda, MD, USA as a category B priority pathogen (2). Consuming undercooked contaminated meat with tissue cysts, ingestion of *T. gondii* oocysts from water, soil, or cat litter and congenital infection through placenta will lead to toxoplasmosis (3–5). Majority of infected individuals are symptoms free (6). *T. gondii* poses a greater risk especially found among pregnant women and immunocompromised individuals. Small percentage of infected newborns develop mild to severe clinical manifestations such as lymphadenopathy, fever and malaise in mild infection, ocular disease and mental illness in moderate manifestation, and severe cases among infected pregnant women will lead to stillbirth, abortion, or live birth children with central nervous system impairment or impaired vision (5). Besides, infected newborns with more virulent types of *T. gondii* may lead to severe and even fatal diseases with pulmonary and multi-visceral involvement (5).

To date, numerous studies have suggested preventive strategies of toxoplasmosis in people having close contact with animals (4, 7, 8), which is due to their high risk behaviors. Unfortunately, scanty data were reported on toxoplasmosis among these people worldwide (9–11). In Malaysia, the seroprevalence of toxoplasmosis in general healthy population increased from 16 to 30% (12). Furthermore, most studies on toxoplasmosis have been mainly conducted in healthy persons, pregnant women, indigenous communities, and HIV-positive patients (12, 13). To the best of our knowledge, this is the first documented data ever reported on toxoplasmosis among animal handlers in Malaysia. In addition, a current situation on epidemiology of toxoplasmosis in animal handlers is crucial and timely to be investigated, so that suggested preventive strategies can be achieved pragmatically in implementation. This study was therefore conducted to determine the seroprevalence of *Toxoplasma* infection among people having close contact with animals and their risk factors in acquiring *Toxoplasma* infection.

## Materials and Methods

### Study site and population

**Figure 1:**
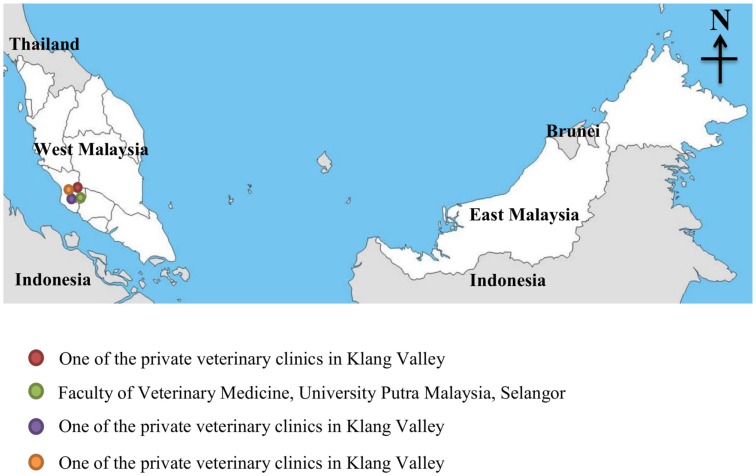
**Study sites in Klang Valley, Malaysia**.

This prospective cross-sectional study was conducted from October 2013 to April 2014. A total of 312 participants were from Faculty of Veterinary Medicine, University Putra Malaysia, Selangor and various private veterinary clinics in the Klang valley (Figure 1) were recruited. The inclusion criteria of this study were (1) immunocompetents who have close contacts with animals which include veterinarians (38), veterinary technicians (45), veterinary students (194), and pet owners (35) and (2) age of more than 15 years. All eligible participants gave informed consent before the commencement of this study. All the participants’ information related to socio-demographic such as their age, education level, occupation, and plausible risk-factors exposure associated with toxoplasmosis (presence of own cats at home, presence of stray cats at home, drinking untreated water, and having contact with soils) prior to 3 months before this study were recorded in the formatted questionnaire forms. An operational definition was used for the risk factors. Presence of own cats at home was defined as a person who is the owner of at least one cat or has close contact with cats while feeding and playing in the house. Presence of stray cats at home was defined as a person having a close proximity with stray cats roaming in the house compound. Drinking untreated water was defined as a person who consumes “untreated water,” e.g., water from a pipe, tap, or rain. Contact with soil (gardening) was defined as person who has a direct exposure to soil while gardening or any kind of outdoor activities.

### Ethical consideration

This study was approved by the ethical review committee of University of Malaya Medical Centre (UMMC), MEC Ref. No. 1024.6 in accordance with the Helsinky Declaration for the inclusion of human subjects in research. The purpose and procedures of this study were explained to all the participants. Informed consents were obtained from agreed participants prior to samples and data collection.

### Serum sample collection

Approximately 5 mL venous blood was drawn, sera were processed and were kept at −20°C until further testing.

### Detection of anti-*Toxoplasma* antibodies

The collected serum was screened primarily for anti-*Toxoplasma* IgG and IgM antibodies by using standard ELISA commercial kit (IgG-NovaLisa, Dietzenbach, Germany) in accordance with the manufacturer’s instruction. A positive sample for the anti-*Toxoplasma* IgG and/or anti-*Toxoplasma* IgM antibody was also tested for its avidity using three standard ELISA commercial kits, namely, ELISA-I, II, and III for comparison and according to its manufacturer instruction. The interpreted results for ELISA-I was that avidity of >40% suggest chronic/past infection and of <40% suggest acute/recent infection, for ELISA-II was that avidity of <15% (low avidity), indicates acute or primary infection and avidity between 15 and 30% (borderline activity) indicates possibility of primary infection during the last 6 months is possible and >30% (high avidity) excludes primary infection within last 3 months and ELISA-III’s avidity of <50% (low avidity) indicates primary infection.

### Statistical analysis

The data collected in the questionnaires and the serology results were analyzed by using statistical software SPSS version 17.0 (SPSS, Inc., Chicago, IL, USA). The qualitative variables were estimated and presented as frequencies and percentages. Univariate analyses and the χ^2^ test were used to investigate the association between *Toxoplasma* seropositivity as a dependent variable and possible demographic and risk factors as independent variables; *p* < 0.05 was regarded as being statistically significant. However, to retain all possible significant association, variables that showed an association with *p* ≤ 0.20 were used to apply to a multivariate logistic regression model (stepwise forward). Each dependent factor was modeled as dichotomous variables.

## Results

During this study period, a total of 312 people were recruited as studied subjects. The age range was 17–64 with a mean 27 ± 9.08 years. Majority of the subjects were in the age group of 21 and 30 years (228; 73.1%), female (234; 75%), Malay (139; 44.6%), veterinary students (194; 62.2%), and city dwellers (300; 96.2%).

The overall seroprevalence of toxoplasmosis in this study was 62 (19.9%) in which 57 (18.3%) samples were positive for IgG, 3 (1.0%) samples were positive for IgM, and 2 (0.7%) samples were positive for both IgG and IgM antibodies (Table 1). The positive IgM antibodies and samples with both positive for IgM and IgG antibodies were further tested for IgG avidity measurement using three standard commercial ELISA kits (I, II, and III) for comparison to differentiate between recent and past infections. Of five *Toxoplasma* seropositive samples, one sample was recently acquired and four other samples were past infections as detected from ELISA-I, all five samples were regarded as recently acquired infection, as demonstrated by ELISA-II, while ELISA-III showed past infection from all five seropositive samples (data were not shown). At the end of this study, there was, however, no clinical evidence of toxoplasmosis diagnosed in the *Toxoplasma* seropositive with low avidity.

**Table 1:** **Seroprevalence of toxoplasmosis among survey population as assessed by the ELISA test**.

ELISA test	*Toxoplasma* seropositivity (62, 19.9%)		
	IgG+ve	IgM+ve	IgG+ve and IgM+ve
Positive	57 (18.3%)	3 (1.0%)	2 (0.6%)
Negative	255 (81.7%)	307 (99.0%)	310 (99.4%)
Total	312	310	312

Univariate analysis in relation to socio-demographic profiles showed that age group, gender, and study population were significantly associated with *Toxoplasma* seropositivity (*p* < 0.05) (Table 2). The results of this study further showed that the highest prevalence of *Toxoplasma* infection was found among veterinary technicians (33.3%) followed by pet owners (31.4%), veterinarians (18.4%), and veterinary students (14.9%). Interestingly, gardening (33; 26.6%) was significantly associated with *Toxoplasma* infection found among these subjects (Table 3). In addition, working duration and task performance were significantly associated with *Toxoplasma* seropositivity found among veterinary personnel (Table 4).

**Table 2:** **Seroprevalence of *Toxoplasma* infection by the demographic characteristics**.

Characteristics	Total *N* = 312	*Toxoplasma* seropositivity *n* (%)	*p*-value
**Age**
Range 17–64 years with a mean of 27 ± 9.08 years		
**Age group**			0.000
≤20	22 (7.1)	3 (13.6)	
21–30	228 (73.1)	36 (15.8)	
31–40	33 (10.6)	9 (27.3)	
≥41	29 (9.29)	14 (48.3)	
**Gender**			0.014
Male	78 (25)	23 (29.5)	
Female	234 (75)	39 (16.7)	
**Race**			0.053
Malay	139 (44.6)	32 (23.0)	
Chinese	115 (36.9)	14 (12.2)	
Indian	33 (10.6)	10 (30.3)	
Aborigine	4 (1.3)	1 (25)	
Foreigner	21 (6.7)	5 (23.8)	
**Study population**			0.011
Veterinarian	38 (12.2)	7 (18.4)	
Veterinary technician	45 (14.4)	15 (33.3)	
Veterinary student	194 (62.2)	29 (14.9)	
Pet owner	35 (11.2)	11 (31.4)	
**Primary residency**			0.233
Village	12 (3.8)	4 (33.3)	
City	300 (96.2)	58 (19.3)	

**p* < 0.05, significant association as potential risk factors; *N*, number examined; *n*, number of positive sample*.

**Table 3:** **Seroprevalence of *Toxoplasma* infection by plausible risk factors among people having close contact with animals**.

Variables	Total *N* = 312	*Toxoplasma* seropositivity *n* (%)	*p*-value
**Close contacts with cats**			0.173
Yes	142	33 (78.6)	
No	170	29 (17.1)	
**Water supply at home**			0.531
River and mountain pipe	27	6 (22.2)	
Government pipe water	283	55 (19.4)	
Private pipe water	2	1 (50)	
**Clean water resources**			0.144
Yes	207	46 (22.2)	
No	105	16 (15.2)	
**Eating with bare hands**			0.379
Yes	233	49 (21.0)	
No	79	13 (16.5)	
**Tasting foods while cooking or seasoning**			0.438
Yes	251	49 (19.5)	
No	61	13 (21.3)	
**Cleaning cooking utensils**			0.054
Yes	300	57 (19.0)	
No	12	5 (41.7)	
**Always gardening**			0.015
Yes	124	33 (26.6)	
No	188	29 (15.4)	

**p* < 0.05, significant association as potential risk factors; *N*, number examined; *n*, number of positive sample*.

**Table 4:** **Seroprevalence of *Toxoplasma* infection by other plausible risk factors in working or study area for veterinary personnel**.

Activities/variables	Total *N* = 277	*Toxoplasma* seropositivity *n* (%)	*p*-value
**Working duration**			0.004
≤1 years	43	6 (14.0)	
2–10 years	218	37 (17.0)	
11–20 years	6	2 (33.3)	
≥21 years	10	6 (60)	
**Task performance**			0.019
Working field	83	22 (26.5)	
Study field	194	29 (14.9)	
**Cleaning cat excrement**			0.286
Yes	217	42 (19.4)	
No	60	9 (15)	
**Wearing gloves**			0.433
Yes	222	40 (18.0)	
No	55	11 (20)	
**Washing hands**			0.665
Yes	275	51 (18.5)	
No	2	0 (0)	

**p* < 0.05, significant association as potential risk factors; *N*, number examined; *n*, number of positive sample*.

Further analysis by multivariate logistic regression showed that age group ≥30 years (OR = 0.34, 95% CI 0.18–0.63) contributes to high *Toxoplasma* seropositivity in the study population and working duration of more than 10 years (OR = 5.07, 95% CI 1.80–14.25) was identified as significant predictors of *Toxoplasma* infection among veterinary personnel (data were not shown).

## Discussion

### Why toxoplasmosis is important

The present study showed the overall seroprevalence of toxoplasmosis among people having close contact with animals was 19.9% and this infection rate did not appear to be very high. Since this is the first study of its kind conducted in Malaysia, it is therefore no previous study can be compared. However, the numbers of respondents provided us substantial interpretation about the current prevalence of toxoplasmosis among these people in Malaysia to signify this conclusive remark. In the literature, the first report on *Toxoplasma* infection among general healthy population in Malaysia was quite low (13.9%) and it has been increasing over the years ranging from 16 to 30% (14), 28.1% (15), and 40.8% (16). However, higher prevalence in the healthy population could be the pet owners and the result may not be comparable with this present study. It is interesting to note that our finding is shown within the same range when compared with (19.9 vs. 14.2%) a rare but similar previous study done in Canada (11). Our study further pointed out that veterinary technicians had the highest *Toxoplasma* infection rate (33.3%). This similar result is shown in the earliest study of toxoplasmosis among veterinary members in the United States (9). In contrary, a previous study from Canada showed that veterinarians had the highest *Toxoplasma* infection rate (16.4%) compared to other groups (11). Based on the results obtained, primary screening of *Toxoplasma* infection should be particularly initiated in high seropositive individuals like veterinary technicians and pet owners. This program should also include women with unknown *Toxoplasma* serostatus to identify primary infection and *Toxoplasma* seronegative individuals for seroconversion. This could help to reduce the incidence in this high risk group of toxoplasmosis.

### When IgG avidity does its role

In the present study, the infection rate of anti-*Toxoplasma* IgM antibodies was 1.0% suggesting a recently acquired *Toxoplasma* infection. Negative results for IgM antibodies strongly exclude the recent infection, while positive result for IgM test is difficult to interpret (10). Hence, the positive result of IgM antibodies was further analyzed using IgG avidity test to help differentiate between past and recent infections (17). Of this, all three seropositive samples for anti-*Toxoplasma* IgM antibodies showed high avidities indicating past infection.

A positive result for only anti-*Toxoplasma* IgG antibodies in this study was 18.3% indicating past or chronic infection. Positive result for both IgG and IgM antibodies in this study was 0.6% indicates either a recent infection or false positive test result (10). Therefore, IgG avidity (brand I, II, and III) measurement, a confirmatory test, was subsequently performed, which is to assist, in determining the time of infection (18, 19). Of this, ELISA-III showed the most accurate result on five seropositive samples indicating chronic or past infection followed by ELISA-II and -I. Supporting to this finding, there was no clinically confirmed case of toxoplasmosis diagnosed during the time of this study. This therefore suggests the following: more than one avidity tests should be performed in a single serum sample, a second blood sample (if no avidity test available) is required to be tested after 2–4 weeks of infection to confirm a recent infection or it can be considered as a false positive with supporting information of whether there is an evidence of clinical toxoplasmosis.

### What background tells its story

Based on demographic data, the seroprevalence of toxoplasmosis was higher in males (28.8%) than females (15.9%). This could be normally explained by the fact that males have a higher tendency to be involved in sports activities or other activities at work or outdoor alike that expose them to soil and also they are not really careful about hand hygiene, which leads to increase risk of acquiring the infection (20). We also found that *Toxoplasma* seropositivity increases with age (21), as also seen the higher infection rate (43.5%) among the older age group (≥41 years old) in this study. This finding is in agreement with previous studies even though studies were conducted in different target groups of population (21, 22). As a multi-racial and -cultural country like Malaysia, it is interesting to note that the highest prevalent rate was found among Indians ethnic (29.0%), which is not surprising because there are outnumber of Indians working closely with animals found in Malaysia compared to other ethnics. This finding is, however, contrary to the fact that the highest seroprevalence of *Toxoplasma* infection has so far been documented among Malay ethnic, which is due to their close contacts with cats (9, 23). Supporting our finding, a previous study in Gombak District, Selangor showed similar result (24). However, ethnic group was not significantly associated with *Toxoplasma* infection in this study.

### How risk factor affects the transmission of *T. gondii*

Our univariate analysis showed that gardening was identified as one of significant risk factors in this study (*p* = 0.015). The participants who frequently do gardening were highly infected (26.6%) compared with the ones who spent less time (15.4%). This could be explained due to the fact that the buried sporulated oocysts of cats might be contaminating the soil the soil and sand and the oocysts remain infectious for about several months and can last beyond 1 year (25). Oocysts have a buoyancy characteristic that may become infectious after raining since oocysts will float on the upper layer of the soil (26). Therefore, it is very important to avoid any materials or foods that come into close contacts with unforeseen contaminated soil. The analysis further showed that the task performance in working field was significantly associated with *Toxoplasma* seropositivity (*p* = 0.019). Of this, veterinarians and veterinary technicians had the higher *Toxoplasma* infection (26.5%) compared to veterinary students (14.9%). This finding was not surprising since the daily task like animal surgery and cleaning the cat excrement are most probably increasing the chance of *Toxoplasma* transmission if the necessary precaution was taken lightly. However, a previous study in Canada demonstrated that cleaning the cat excrement was not the significant factor contributing to *Toxoplasma* infection among veterinary personnel (11). Based on this finding, other unidentified risks associated with *Toxoplasma* infection should be further investigated before any conclusion could be made. Of note, working duration was also significantly associated with *Toxoplasma* infection (*p* = 0.004) where the longest working duration of ≥21 years had the highest prevalent rate (60%). This might be due to the higher exposure with the animals especially cats since they have a lot of experience in handling and having close contact with cats.

After multivariate logistic regression model was applied, it was interesting to find that only age group of ≥30 years old and working duration of >10 years were identified as significant risks for *Toxoplasma* infection. This could be explained due to the fact that primary behavioral practices should be advised among people with increasing age, which is more prone to disease transmission of *T. gondii*. Also, individual with increasing age may have other co-infections that may lower his immune system, which can increase the susceptibility to *Toxoplasma* infection. The longer working duration contributed to the risk factor of *Toxoplasma* acquisition since they had a daily routine of handling with animals for long hours. This suggests that they might have a higher chance of close contact with cats and more likely expose to sporulated oocysts in cat’s feces, which can increase the risk of *Toxoplasma* transmission (11).

## Conclusion

This preliminary study shows the high prevalence of chronic toxoplasmosis in both veterinary personnel and pet owners. Age group of ≥30 years old in overall studied populations and working duration of >10 years among veterinary personnel significantly contributed to *Toxoplasma* infection. Hence, basic personal hygiene and management in working and study areas among veterinary personnel should be taken into consideration to minimize the probability of exposure to *Toxoplasma* infection. Future similar study is recommended periodically and also to investigate other unidentified risk factors to eliminate the infection rate and to eradicate this parasite from the region.

This work was presented in part at the ICOPA XIII, Mexico, 10–15 August, 2014.

## Author Contributions

VN and TTC designed the study. NAA, TTC, RSKS, and VN carried out the study. GJBM contributed most on manuscript writing. RSKS, YALL, TTC, HA, and VN helped in manuscript writing and editing. RSKS, YALL, TTC, and VN provided opinions and suggestions about this manuscript. All authors read and approved the final version of the manuscript.

## Conflict of Interest Statement

The authors declare that the research was conducted in the absence of any commercial or financial relationships that could be construed as a potential conflict of interest. The Review Editor Yeng Chen declares that, despite being affiliated to the same institution as authors Guo-Jie Brandon-Mong, Nurul Asma Anati Che Mat Seri, Hemah Andiappan, Tian-Chye Tan, Yvonne Ai-Lian Lim and Veeranoot Nissapatornand, the review process was handled objectively and no conflict of interest exists.
